# Patient Engagement With a Mobile Web-Based Telemonitoring System for Heart Failure Self-Management: A Pilot Study

**DOI:** 10.2196/mhealth.3789

**Published:** 2015-04-01

**Authors:** Shiyi Zan, Stephen Agboola, Stephanie A Moore, Kimberly A Parks, Joseph C Kvedar, Kamal Jethwani

**Affiliations:** ^1^Center for Connected HealthPartners HealthCareBoston, MAUnited States; ^2^Harvard Medical SchoolBoston, MAUnited States; ^3^Massachusetts General HospitalBoston, MAUnited States

**Keywords:** heart failure, disease self-management, remote monitoring, telemonitoring, interactive voice response system, mobile health, Web portal, patient engagement, quality of life

## Abstract

**Background:**

Intensive remote monitoring programs for congestive heart failure have been successful in reducing costly readmissions, but may not be appropriate for all patients. There is an opportunity to leverage the increasing accessibility of mobile technologies and consumer-facing digital devices to empower patients in monitoring their own health outside of the hospital setting. The iGetBetter system, a secure Web- and telephone-based heart failure remote monitoring program, which leverages mobile technology and portable digital devices, offers a creative solution at lower cost.

**Objective:**

The objective of this pilot study was to evaluate the feasibility of using the iGetBetter system for disease self-management in patients with heart failure.

**Methods:**

This was a single-arm prospective study in which 21 ambulatory, adult heart failure patients used the intervention for heart failure self-management over a 90-day study period. Patients were instructed to take their weight, blood pressure, and heart rate measurements each morning using a WS-30 bluetooth weight scale, a self-inflating blood pressure cuff (Withings LLC, Issy les Moulineaux, France), and an iPad Mini tablet computer (Apple Inc, Cupertino, CA, USA) equipped with cellular Internet connectivity to view their measurements on the Internet. Outcomes assessed included usability and satisfaction, engagement with the intervention, hospital resource utilization, and heart failure-related quality of life. Descriptive statistics were used to summarize data, and matched controls identified from the electronic medical record were used as comparison for evaluating hospitalizations.

**Results:**

There were 20 participants (mean age 53 years) that completed the study. Almost all participants (19/20, 95%) reported feeling more connected to their health care team and more confident in performing care plan activities, and 18/20 (90%) felt better prepared to start discussions about their health with their doctor. Although heart failure-related quality of life improved from baseline, it was not statistically significant (*P*=.55). Over half of the participants had greater than 80% (72/90 days) weekly and overall engagement with the program, and 15% (3/20) used the interactive voice response telephone system exclusively for managing their care plan. Hospital utilization did not differ in the intervention group compared to the control group (planned hospitalizations *P*=.23, and unplanned hospitalizations *P*=.99). Intervention participants recorded shorter average length of hospital stay, but no significant differences were observed between intervention and control groups (*P*=.30).

**Conclusions:**

This pilot study demonstrated the feasibility of a low-intensive remote monitoring program leveraging commonly used mobile and portable consumer devices in augmenting care for a fairly young population of ambulatory patients with heart failure. Further prospective studies with a larger sample size and within more diverse patient populations is necessary to determine the effect of mobile-based remote monitoring programs such as the iGetBetter system on clinical outcomes in heart failure.

## Introduction

### The Burden of Congestive Heart Failure

Congestive heart failure is a chronic condition that is associated with significant morbidity, mortality, and reductions in quality of life, particularly among older adults ≥ 65 years of age. Hospital readmission rates for heart failure are among the highest of any chronic disease, and account for much of the financial burdens on the health care system. Upon discharge from the hospital, half of heart failure patients experience rehospitalization within 6 months [1]. In 2009, the total cost of heart failure-related treatment in the United States was about US $39 billion; by 2030, this number is projected to double as a result of our aging population [2].

### Remote Monitoring for Congestive Heart Failure

Remote monitoring by structured telephone support or telemonitoring has been commonly explored as a promising strategy for improving heart failure outcomes [3]. These programs offer the potential to provide access to specialist care for a much larger number of patients across a much greater geography, assist health care providers in patient management, and effectively lower the burden of care from providers by engaging and supporting patients in self-care practices [3]. Nevertheless, to date, remote monitoring programs that have achieved success in improving clinical outcomes and reducing hospital readmissions are highly intensive (ie, requiring close clinical oversight and follow-up), and may therefore not be appropriate for all patient groups, especially those who have less severe disease. Furthermore, the scalability of remote monitoring programs has often been limited by high equipment costs and the logistics and time delay associated with initial patient set-up on these programs. Considering that a high proportion of 30-day readmissions occur relatively soon after patients are discharged, there is a need for a solution that can help facilitate smoother transitions in care from the hospital to the home environment [4].

It is widely accepted in the heart failure literature that patients who are actively involved in their own care and adhere to treatment regimens are more likely to have improved survival, decreased readmission rates, and experience better quality of life [5,6]. The nearly ubiquitous penetration of wireless Internet, adoption of mobile phones, and availability of portable and affordable consumer-facing personal health monitoring devices offer a potential means of addressing the demands and burdens associated with disease self-management, while reducing heart failure-associated health care costs. To date, a number of studies have evaluated mobile device-based telemonitoring programs for heart failure, but no existing system that we are aware of has incorporated a patient-facing Web portal and leveraged consumer-facing digital devices to engage and empower patients in disease self-management [7-10].

### Our Aims

In this study, we pilot-tested a Web- and telephone-based self-management intervention that leverages personal digital health monitoring devices in a population of ambulatory, adult heart failure patients. Our primary aim was to evaluate the feasibility and acceptability of use of the intervention in patients with heart failure. In addition, we assessed patient engagement with the program, and its impact on patients’ quality of life. We also examined the effect of the intervention on hospital utilization by comparing patients who used the program with a matched set of passive controls obtained from the electronic medical record (EMR) system.

## Methods

### Study Design

This feasibility pilot was designed as a single-arm prospective study in which participants used the intervention for heart failure self-management over a 90-day study period.

### Study Participants

Participants were recruited from the outpatient clinic of two cardiologists of the Massachusetts General Hospital (MGH) Heart Center’s Heart Failure and Cardiac Transplant Program. As this was a pilot study, a convenience sample was used, consisting of patients who had scheduled outpatient visits over the course of a 4-month period between February and May 2013.

Patients were deemed eligible if they were ambulatory, English-speaking adults ≥18 years of age with a current diagnosis of heart failure. Patients were required to have regular access to a telephone and be able to navigate a simple website, understand the scope of the study, and provide written informed consent. Patients who were admitted to the hospital and/or enrolled in another remote monitoring program, as well as those with significant visual, hearing and/or cognitive impairments, and those with significant medical or psychiatric comorbidities were excluded. The Partners HealthCare Institutional Review Board approved all study procedures.

### Study Procedures

After obtaining informed consent at an initial study visit, patients were set up with an account on the Web platform (their own “patient” portal) and were trained on how to use all components of the intervention.At the visit, demographic and other baseline data were collected using an enrollment questionnaire and digital devices were provided as part of the study. In addition to baseline demographics, the questionnaire also captured patients’ baseline technology use and baseline social health using the emotional, informational, and instrumental support domains of the Patient Reported Outcomes Measurement Information System measures, and heart failure-related quality of life using the Minnesota Living with Heart Failure Questionnaire (MLHFQ). Given that depression is a known risk factor of poor outcomes in heart failure, in addition, we also assessed levels of baseline depression using the 8-item Patient Health Questionnaire (PHQ-8) [11].

Patients were provided with a step-by-step reference guide, and instructed to take their measurements and log their care plan activities daily through the patient portal and/or the interactive voice response (IVR) telephone system. They were also instructed to notify study staff in advance if they expected any interruptions in study procedures (ie, planned hospital admissions) so that their study timeline could be adjusted appropriately. Following completion of the 3-month study, patients completed a closeout questionnaire by mail. All other relevant study data were collected from the EMR system and the Web platform.

Matched controls were identified from the EMR system, via the Partners HealthCare Research Patient Data Repository, for comparison with study participants on hospital resource utilization. Patients receiving the intervention were matched 1:1 with controls by age (±2 years), gender, race, and diagnosis.

### Study Intervention

The iGetBetter system is comprised of a Web platform, IVR system, and portable consumer-facing digital devices that measure and collect key vital signs. Patients were enrolled in the intervention following a regular scheduled outpatient appointment with their cardiologist at the MGH Heart Failure Clinic, and began using the system at home the following morning.

**Figure 1:**
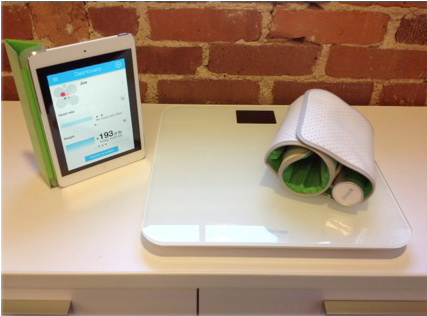
Portable devices provided to participants as part of the intervention (from left: iPad Mini, bluetooth weight scale, auto-inflating blood pressure cuff).

Patients were instructed to take their weight, blood pressure, and heart rate measurements each morning using the provided devices (see Figure 1), which included a WS-30 bluetooth weight scale and self-inflating blood pressure cuff (Withings LLC, Issy les Moulineaux, France). Patients were also provided with an iPad Mini tablet computer (Apple Inc, Cupertino, CA, USA) equipped with cellular Internet connectivity for the accessibility and convenience of being able to view their measurements online.

**Figure 2:**
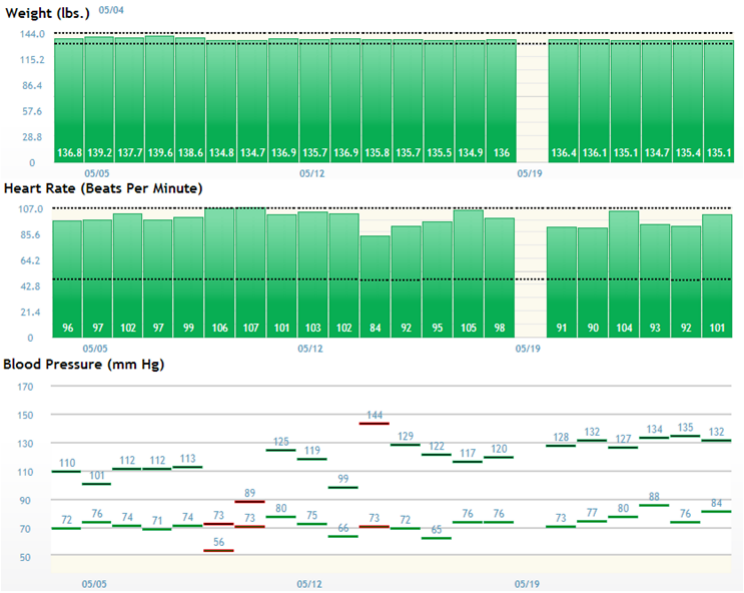
Screenshot of a sample progress report on the Web portal displaying a graphical representation of a patient’s vitals taken using study devices.

**Figure 3:**
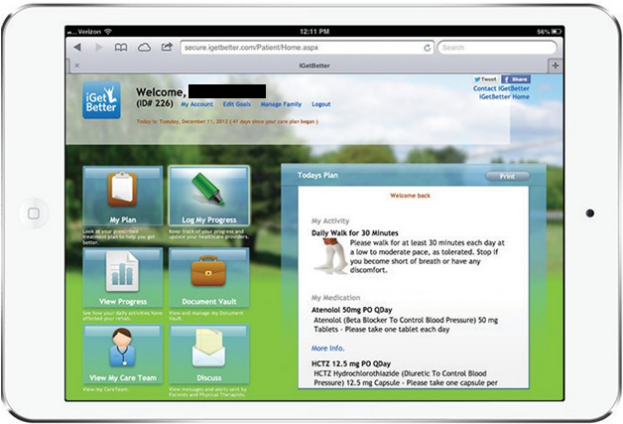
Screenshot of the patient-facing Web portal upon logging in.

Measurements taken by patients were transmitted onto the Web platform, where they were stored and displayed in a graphical fashion (see Figure 2). Patients were instructed to log into the patient portal (see Figure 3) to check off their care plan activities using the iPad Mini provided or their own computer at home. Patients who did not complete one or more of the listed steps prior to their self-selected morning reminder call time would receive a reminder phone call from the IVR system prompting them to log their care activities and/or manually enter in their measurements using their phone keypad. The IVR system essentially provided patients an alternative, manual means of recording their measurements and care activities, which were similarly uploaded and stored on the centralized Web platform.

Study participants’ cardiologists had access to the Web platform through a “clinician” portal to view their patients’ progress over time, but were not required to perform any active role in the study aside from recommending a predefined range of acceptable values for vital signs, which were input into the system for each patient during enrollment. During the study, if a patients’ measurement fell outside of the preset range, a system alert would be triggered; research study staff monitored the Web platform for these alerts during regular business hours. Study staff made follow-up calls to patients if their measurements fell outside of their predefined range, and all clinically relevant alerts were routed to a patient’s cardiologist and their care team. We emphasized to patients that the system was not to be used for reporting emergencies and did not serve as a substitute to their usual care regimen and clinic visits.

### Outcome Measures

#### Primary Outcome

Usability and satisfaction with the intervention was assessed through self-reported patient questionnaires.

#### Secondary Outcomes

Hospital resource utilization encompassed visits to the emergency room/urgent care clinic, as well as heart failure-related inpatient hospitalizations. The primary source of data for this measure was the EMR system for both the intervention group and their matched controls, but the intervention group was asked to self-report any additional out-of-system hospitalizations in the study closeout questionnaire. Heart failure-related quality of life was assessed pre and postintervention using the MLHFQ [12]. Previous studies have shown this questionnaire to be sensitive to quality of life changes in the heart failure patient population [13,14]. Engagement with the intervention was assessed objectively via daily care plan logging and Web portal log-ins.

### Data Analysis

Baseline characteristics for the intervention group and EMR-matched control group were summarized with percentages for categorical variables and means and SD for continuous variables. The frequency and duration of hospitalizations for patients receiving the intervention were summarized and compared with EMR-matched control data. Descriptive statistics were used to analyze and summarize usability and satisfaction measures, heart failure-related quality of life measures, and overall engagement indices. Data analysis was performed using Stata version 12 with an alpha of .05 set *a priori*.

## Results

### Study Recruitment and Baseline Characterisitics

**Figure 4:**
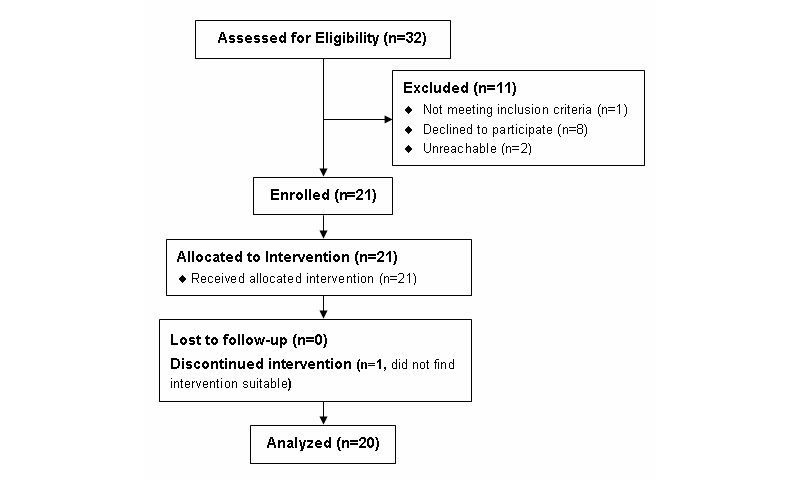
Participant enrollment and inclusion in to the study.

Of 32 patients assessed for eligibility, 21 agreed to participate and were enrolled into the study. There were 11 patients that were excluded from the study: 1 did not meet eligibility criteria, as they were no longer receiving care from an MGH cardiologist; 8 declined to participate due to either transportation limitations or lack of interest in the study; and 2 were unreachable (see Figure 4). Of the 21 enrolled, 20 completed the study.

The mean age of study participants was 53 years (SD 17); these patients were predominantly male, white, married, and had received higher education (ie, > 1 year of college-level education). One-third of participants were employed full-time at the time of enrollment (see Table 1). EMR-matched control demographics were similar to that of study participants and are reported in Table 1.

Of the 21 enrolled participants, 11 (52%) reported no depression as measured by the PHQ-8, while 3 (14%) reported moderate-severe levels of depression (see Table 1). The vast majority of participants scored well on social health; all reported adequate informational support, while the mean scores for emotional support and instrumental support were 96% and 94%, respectively. Participants’ baseline technology use is displayed in Table 2.

**Table 1:** Baseline demographic characteristics of study participants.

Baseline demographic characteristics		Intervention group, N=21	Matched control group, N=20
**Age, years (SD)**			
	Mean	53 (17)	53 (17)
	Range	21-81	22-81
Average # in household		2.4	-
Male gender, n (%)		15 (71)	14 (70)
**Marital status, n (%)**			
	Married	19 (90)	7 (35)
	Single	1 (5)	6 (30)
	Divorced	1 (5)	2 (10)
**New York Heart Association class, n (%)**			
	1	5 (24)	-
	2	9 (43)	-
	3	7 (33)	-
Left ventricular ejection fraction, mean (SD)		34.6 (14.9)	-
**Education, n (%)**			
	4+ yrs of college	12 (57)	-
	1-3 yrs of college	3 (14)	-
	12th grade, GED	3 (14)	-
	9th-11th grade	1 (5)	-
	1st-8th grade	1 (5)	-
**Race, n (%)**			
	White	19 (90)	16 (80)
	Black/African American	2 (10)	2 (10)
	Other	0 (0)	2 (10)
**Employment status, n (%)**			
	Employed full-time (includes self-employment)	7 (33)	-
	Retired	7 (33)	-
	Disabled	4 (19)	-
	Employed part-time (includes self-employment)	1 (5)	-
	Homemaker	1 (5)	-
	Unemployed	1 (5)	-
**PHQ-8, n (%)**			
	None (0-4)	11 (52)	-
	Mild (5-9)	5 (24)	-
	Moderate (10-14)	2 (10)	-
	Moderate-severe (15-19)	3 (14)	-
	Severe (20-24)	0 (0)	-
**Social support (mean score %)**			
	Emotional	96	-
	Informational	100	-
	Instrumental	94	-

**Table 2:** Baseline technology use of study participants (N=21).

Baseline technology use		n (%)
**Method of accessing the Internet**		
	Broadband	12 (60)
	Cellular network	10 (50)
	Wireless network	10 (50)
	Dial-up telephone	2 (10)
**Have used the Internet for...**		
	Email	19 (95)
	Looking for health/medical information	17 (85)
	Banking	17 (85)
	Sharing photos	16 (80)
	Instant messaging/online chat	15 (75)
	Accessing social networking sites	9 (45)
	Tracking weight, diet, or exercise routine	4 (20)
	Tracking other health indicators (eg, blood pressure, sleep, headaches)	4 (20)
**Computer ownership**		
	Laptop computer	15 (75)
	Desktop computer	14 (70)
	Tablet computer	8 (40)
**Telephone ownership**		
	Landline phone	15 (75)
	Cellular phone	17 (85)
	Mobile phone	15 (75)
**Have used a cellular phone for...**		
	Text messaging	16 (80)
	Sharing photos	15 (75)
	Accessing the Internet	14 (70)
	Email	14 (70)
	Banking	12 (60)
	Looking for health/medical information on the Internet	10 (50)
	Accessing social networking sites	6 (30)
	Tracking weight, diet, or exercise routine	3 (15)
	Tracking other health indicators (eg, blood pressure, sleep, headaches)	2 (10)

### Usability and Satisfaction With the Intervention

Overall, all 20 study participants reported high levels of satisfaction with the intervention. Of these participants, 19 (95%) agreed “mostly” if not “definitely” with statements evaluating whether they felt comfortable using the intervention, whether they were satisfied with how easy it was to use the intervention, and whether they would recommend the intervention to friends and family members (see Table 3).

All participants also rated the intervention highly on usability, with the majority expressing definite agreement to statements regarding how easy it was to learn the system and carry out their home care activities using the system (see Table 4).

**Table 3:** Overall usability and satisfaction with the intervention as reported by study participants (N=20).

Survey question	Likert response, n (%)			
	Definitely true	Mostly true	A little bit true	Definitely not true
I felt comfortable using this system	18 (90)	2 (10)		
I was able to easily perform my home care activities using this system	16 (80)	4 (20)		
It was easy learning to use this system	15 (75)	4 (20)	1 (5)	
I am satisfied with how easy it was to use this system	13 (65)	7 (35)		
I would recommend the system to a friend or family member	12 (60)	7 (35)	1 (5)	

**Table 4:** Usability and satisfaction with specific components of the intervention as reported by study participants (N=20).

Survey question	Likert response, n (%)			
	Definitely true	Mostly true	A little bit true	Definitely not true
The measurement devices were easy to use	14 (70)	6 (30)		
The iPad Mini was easy to use	14 (70)	5 (25)	1 (5)	
It was easy to enter my data and log care plan activities on the website	7 (35)	6 (30)	2 (10)	3 (15)
The alert function was very useful when my measurements were out of range	7 (35)	4 (20)	3 (15)	4 (20)

### Perceived Effect of the Intervention on Care

All but one of the final 20 participants (95%) reported feeling more confident in performing home care activities, and more connected to their health care team. Of these participants, 18 (90%) felt that the system helped them in starting discussions about their health with their doctor, and 16 (80%) also believed that their disease was better controlled as a result of using the intervention (see Table 5).

**Table 5:** Patients’ perceived effect of the intervention on heart failure related care (N=20).

Survey question		Likert response, n (%)			
		Definitely true	Mostly true	A little bit true	Definitely not true
**The intervention helped me...**					
	Feel more confident in performing my home care activities	12 (60)	5 (25)	2 (10)	1 (5)
	Feel more connected to my care team	10 (50)	4 (20)	5 (25)	1 (5)
	Start discussions about my health with my doctor	8 (40)	4 (20)	5 (25)	2 (10)
	Better control my disease	6 (30)	7 (35)	3 (15)	4 (20)
	Remember to take my medications more regularly	8 (40)	1 (5)	5 (25)	6 (30)

### Feedback on Intervention Components

All participants “liked” the measurement devices (ie, blood pressure cuff and weight scale) that were part of the intervention, and reported that they found the devices easy to use. With respect to the telephone component, 7 of the 20 participants (35%) “liked” the automated IVR reminder phone calls, and 14 (70%) reported that they found the follow-up phone calls from study staff helpful whenever they recorded an out-of-parameter measurement (see Table 6).

**Table 6:** Participants’ (N=20) ratings of statements regarding use of the intervention components.

Survey question	Likert response, n (%), N=20			
	I like it very much	I like it	I like it a little bit	Not at all
Checking weight	13 (65)	5 (25)	2 (10)	
Blood pressure monitoring	12 (60)	6 (30)	2 (10)	
Out-of-range alerts for measurements	8 (40)	5 (25)	1 (5)	5 (25)
Viewing measurements on the website	7 (35)	3 (15)	5 (25)	3 (15)
IVR reminder phone calls	3 (15)	1 (5)	3 (15)	13 (65)

### Use of the Web Platform

Of the 20 participants, 11 (55%) reported that they logged in to the patient portal to view their measurements. Of the 11 individuals, 7 (64%) viewed their measurements daily, and 8 (73%) believed that being able to view their measurements inspired more interest in their own health, and helped them better manage their health condition. All but one of the 11 participants (91%) reported discussing the system with others, while only 6 (55%) reported discussing the system with their doctor.

### Engagement With the Intervention

The overall engagement with the two main components of the intervention (ie, the IVR system and the Web platform) was assessed by participants’ daily care plan logging trends. Over half of study participants had 80% or greater adherence to care plan logging over the course of the study (ie, logged data for more than 72/90 days). Although most participants engaged through the patient portal, 3 participants (15%) who displayed high adherence to care plan logging were found to have used the IVR system exclusively. Upon breaking down patient engagement with the intervention by study week, we found that overall care plan logging engagement decreased following the first 4 weeks, but 15 of the 20 participants continued to engage with care plan logging throughout each week of the study.

The median number of Web portal log-ins was 28 log-ins per day in the first week of the study; log-ins decreased in subsequent weeks, but remained above 12 log-ins per day for the duration of the study period. Although 4 participants (20%) were found to have stopped logging in to their patient portal after the first week, 14 (70%) appeared to have consistently accessed the patient portal for the duration of the study.

### Hospital Resource Utilization

The breakdown of all hospital encounters for the intervention group and EMR-matched control group are displayed in Table 7. Within the intervention group of 20 participants, 2 patients (10%) recorded unplanned hospital visits, and 3 (15%) recorded planned hospital admissions during the study. Only one of these hospital encounters was found to be a 30-day readmission. In addition, 3 patients (15%) from the intervention group were admitted to the hospital for planned procedures during the study period. Within the EMR-matched control group, one patient (5%) recorded hospitalizations within the study timeframe; this individual was found to have been hospitalized 3 times, and recorded two 30-day readmissions (see Table 7).

The mean unplanned length of hospital stay for the intervention group was 3.4 days compared to 8.7 days in the EMR-matched control group, although no significant differences were observed between the two groups (*P*=.30). Overall, no significant differences were observed between the intervention group and control group in the number of admissions, length of hospital stay, or frequency of 30-day readmissions.

**Table 7:** Aggregate hospital encounter data for study participants and EMR-matched controls.

		Intervention, N=20	Control, N=20	*P* value
**Inpatient hospital admissions, # of encounters (# of patients)**				
	Planned	5 (3)	0	.23
	Unplanned	2 (2)	3 (1)	.99
Emergency room/urgent care visits, # of encounters (# of patients)		2 (2)	1 (1)	.99

### Change in Self-Reported Quality of Life and Health

On the MLHFQ, a lower score indicates improvement in quality of life. Study participants’ score on the MLHFQ decreased by approximately 4 points from pre to postintervention (see Table 8). This difference did not reflect a clinically meaningful change, and was not found to be statistically significant (*P*=.55).

Participants’ score on the general self-rated health question item increased by 0.05 points from pre to postintervention, but this difference was not found to be statistically significant (*P*=.83) (see Table 8).

In addition to measures of heart failure-related quality of life, patients were also asked validated question items evaluating one’s confidence in their ability to perform home care activities, take medications, and attend scheduled medical appointments. We found that the responses of patients to these questions did not change as a result of the intervention, and no statistically significant changes were detected.

**Table 8:** Quality of life measures pre and postintervention for study participants (N=20).

Self-reported health measure		Preintervention score, mean (SD)	Postintervention score, mean (SD)	Difference, mean (SD)	*P* value
**Minnesota Living with Heart Failure Questionnaire Scale**					
	Overall	43 (26)	39 (27)	-4 (31)	.55
	Physical	19 (12)	17 (13)	-2 (13)	.46
	Emotional	9 (7)	8 (7)	-1.7 (9.0)	.44
**General self rated health item**					
	Overall	2.70 (1.22)	2.75 (1.21)	0.05 (1.05)	.83

## Discussion

### Principal Findings

We report the results of a formative evaluation of a Web- and telephone-based intervention, leveraging portable consumer health technologies, for low intensive heart failure self-monitoring in a population of ambulatory, adult heart failure patients recruited from the cardiology clinic of an academic medical center. To the best of our knowledge, this is the first study evaluating the use of such a program incorporating portable consumer-facing digital devices to engage heart failure patients in self-managing their disease outside of the hospital setting.

Overall, patients reported high acceptability of the iGetBetter system, and found the intervention highly feasible and applicable to their care. Although the intervention encouraged patients to take their vitals, which were automatically transferred to a centralized Web portal, we also offered patients the option of using an IVR telephone system to report data to the Web platform (albeit by manual input of measurements), and set automated daily reminder calls for their care plan items. Our most significant finding was that 80% of patients (16/20) maintained a consistent pattern of reporting and viewing their data over the course of the 90-day follow-up period. In contrast, other studies have found engagement levels to be lower, and also taper off over time [7-10,15]. A possible explanation for our observed findings is that the portability and user-friendliness of study devices played an important role in fostering patient engagement compared to often outdated conventional remote monitoring devices (which rely on telephone lines to transmit data) that have been used in previous studies.

To gain further understanding of patients’ use of the system, we examined the means by which patients engaged with different components of the intervention. In doing this, we found that 55% of study participants (11/20) consistently logged in to their patient portal to view their data; and that among these individuals, 64% (7/11) logged in using the iPad Mini provided and viewed their data daily. In addition, 73% of these patients (8/11) believed that being able to view their measurements inspired more interest in their own health, and that this in turn helped them better manage their health condition. These numbers show that despite low overall engagement, all patients who did engage appeared to have reported some benefit as a result of using the system. Furthermore, although only 35% of patients (7/20) reported finding the IVR system helpful, 15% (3/20) continued to use the IVR to log their data during the study. Given that the majority of the population today owns a mobile phone and mobile phone adoption rates are on the rise in older adults, the development of a Web platform that is compatible across multiple devices including a mobile phone interface may prove to be more useful and engaging for a wider spectrum of patient populations [16].

With respect to hospital resource utilization, although the total number of unplanned inpatient and emergency room/urgent care visits was comparable between the two groups, the mean duration of hospital stay for unplanned admissions in the EMR-matched control group was over twice as long as that of patients using the intervention. Furthermore, patients using the intervention also recorded fewer 30-day readmissions than controls, although these differences were not statistically significant. Nevertheless, a number of patients visited the hospital for scheduled procedures during the study period. It is possible that as a result of being more connected with their care team, the intervention helped facilitate better communication between patients and their care providers, resulting in more timely care management decisions. It is also possible that the ability to view patients’ physiologic trends over time, in addition to alert notifications, could have provided clinicians with more data that allowed them to determine when an intervention or follow-up with a patient was warranted. In accordance with our findings, a previous study by Wu et al has also reported trends toward increased hospitalizations for planned procedures among users of a Web-based heart failure management platform compared to nonusers receiving standard care [17].

Quality of life, a major issue in heart failure and a key focus of treatment, is affected by the functional capabilities, symptoms, and psychosocial perceptions of patients [5]. Although not statistically significant, the final quality of life measure improved from baseline, suggesting that the intervention may have had a positive effect on patients’ self-perceived disease burden. Eighty-percent of patients (16/20) believed that their heart failure was better controlled as a result of using the intervention; this, along with other feedback we received from patients, suggests that the majority felt more empowered to carry out their self-care activities at home with the help of the system. Although more impressive and significant improvements in quality of life have been observed in studies using Web-based heart failure self-management programs, in our study, we enrolled patients in the outpatient setting while they were clinically stable, and patients were also only followed up for a 90-day study period, so it is possible that observing a change in quality of life under these circumstances was more difficult [15,18,19].

The fact that over half of our study population had greater than 80% adherence to daily care plan logging throughout the study period is encouraging and somewhat unprecedented when compared with previous Web-based self-management programs for heart failure. Nevertheless, a few patients did not engage with the system for reasons that may range from a lack of insight regarding their condition, not being sufficiently “activated” for self-management, or potentially, if they were already satisfied with their level of knowledge regarding self-care.

### Limitations

A limitation to our study is in the small sample size. Because we designed the study as a feasibility pilot, we did not conduct a formal sample size calculation, and our study was not powered to detect the effects of the intervention on clinical outcomes. Additionally, since patients were identified through purposeful sampling from two cardiologists to participate in the study, selection bias cannot be ruled out. Although one patient reported not having an Internet connection at home, the majority of patients were more likely to be familiar with technology at baseline; it may well be the case that patients who may not have been as tech savvy, but who were at least more open to trying out a new program were more likely to enroll and engage with the system. Furthermore, those who comprised our study sample were predominantly married, white male, ambulatory heart failure patients with varying socioeconomic and educational backgrounds; this limits the generalizability of our findings and precludes us from extrapolating findings from this study to other populations. Finally, due to the relatively low intensity of the remote monitoring component, the current system may not be suitable for patients with more severe disease; further investigation involving a more diverse heart failure patient population with varying degrees of disease severity is necessary to evaluate the true impact of the intervention.

### Conclusions

Our study demonstrates the feasibility and acceptability of a Web-based telemonitoring system that incorporates personal digital health monitoring devices in a group of ambulatory heart failure patients. Although this study is a small sample pilot not powered for statistical inference, our findings suggest the potential for improved health outcomes in similar patient populations who use the system. The portability and convenience offered by the consumer-facing digital devices provided as part of the remote monitoring system likely contributed to patient satisfaction and high engagement levels, while dissatisfaction with the IVR system and technical difficulties likely affected adoption and engagement in certain patients. Further study within a larger sample size is needed to determine the extent of the benefits of the system on heart failure outcomes.

## Abbreviations

EMR: electronic medical record
IVR: interactive voice response
MGH: Massachusetts General Hospital
MLHFQ: Minnesota Living with Heart Failure Questionnaire
PHQ-8: Patient Health Questionnare-8
